# Mycorrhizae and Rhizobacteria on Precambrian Rocky Gold Mine Tailings: II. Mine-Adapted Symbionts Alleviate Soil Element Imbalance for a Better Nutritional Status of White Spruce Seedlings

**DOI:** 10.3389/fpls.2018.01268

**Published:** 2018-09-03

**Authors:** Martin B. Nadeau, Joan Laur, Damase P. Khasa

**Affiliations:** ^1^Viridis Terra Innovations Inc., Sainte-Marie, QC, Canada; ^2^Institut de Recherche en Biologie Végétale, Université de Montréal, Montréal, QC, Canada; ^3^Centre for Forest Research and Institute of Integrative and Systems Biology, Université Laval, Quebec City, QC, Canada

**Keywords:** mycorrhizae, land reclamation, mine waste, nutrition, *Picea glauca*, rhizobacteria, toxicity

## Abstract

In the context of a phytorestoration project, the purpose of this study was to assess the respective contribution to the nutritional status of *Picea glauca* seedlings of ectomycorrhizae and rhizobacteria native or not to the Sigma-Lamaque gold mine wastes in northern Quebec, Canada. In a glasshouse experiment, inoculated plants were grown for 32 weeks on coarse waste rocks or fine tailings obtained from the mining site. The survival, health, growth, and nutritional status of plants were better on coarse waste rocks than on fine tailings. Fe and Ca were especially found at high levels in plant tissues but at much lower concentrations on waste rocks. Interestingly, inoculation of microsymbionts had only minimal effects on N, P, K, and Mg plant status that were indeed close or within the concentration range encountered in healthy seedlings. However, both fungal and bacterial treatments improved Fe and Ca concentrations in plant tissues. Fe concentration in the foliage of plants inoculated with the fungi *Tricholoma scalpturatum* Tri. scalp. MBN0213 GenBank #KC840613 and *Cadophora finlandia* Cad. fin. MBN0213 GenBank #KC840625 was reduced by >50%. Both fungi were isolated from the mining site. The rhizobacteria, *Azotobacter chroococcum*, also improved plant Fe level in some cases. Regarding Ca nutritional status, the native bacterial strain *Pseudomonas putida* MBN0213 GenBank #AY391278 was the only symbiont that reduced foliar content by up to 23%. Ca concentration was negatively correlated with the fungal mycorrhization rate of seedling roots. This relation was especially strong (*r* = -0.66, *p*-value ≤ 0.0001) in the case of *C*. *finlandia*. Also, a similar relationship existed with root Fe concentration (*r* = -0.44, *p*-value ≤ 0.0001). In fact, results showed that seedling performance was more correlated with elevated Ca and Fe concentration *in planta* than with nutrient deficiency. Also, native microsymbionts were capable of regulating seedling nutrition in the poor substrate of the Sigma-Lamaque gold mine tailings.

## Introduction

Anthropogenic activities such as mining of the Precambrian gold ores create severely disturbed ecosystems: most nutrients and minerals are trapped in the rock tailings, the only soil left. The situation is very similar to the natural process that occurs after glaciation retreat (Taner et al., 1986; Balogh-Brunstad et al., 2008) – yet, over time, many species recolonize this low fertility environment where organic matter is inexistent (Hobbie et al., 1998). Should it be cautiously designed, phytorestoration is an appropriate on-site management strategy for the reclamation of mine residues. Phytoremediation is an efficient, economically and ecologically sound solution (Nadeau, 2014; Vodouhe and Khasa, 2015).

Jumpponen et al. (2002) have studied the occurrence of ectomycorrhizae (ECM), the first mycorrhizal fungi present in primary succession (Trowbridge and Jumpponen, 2004), on the forefront of a retreating glacier. They found that pioneer plants were only able to thrive on the rock tailings in association with ECM. Like alder or spruce trees, all studied species (*Salix commutata, S*. *phylicifolia, Abies lasiocarpa, Larix lyallii, Pinus contorta*, and *Tsuga mertensiana*) are commonly known to form mycorrhizal symbiotic relationships (Dixon and Buschena, 1988; Nienstaedt and Zasada, 1990; Dahlberg, 2001; Quoreshi et al., 2007; Roy et al., 2007; Smith and Read, 2008). To thrive in harsh post-glacial conditions or human-made new ecosystems, plants have co-evolved with their microsymbionts capable of scavenging nutrients from rocks or fixing atmospheric nitrogen in exchange for plant photosynthetic carbon sources (Allen et al., 2003a,b; Khan, 2006; Roy et al., 2007; Quoreshi and Khasa, 2008).

One of the most characteristic trees of the boreal forest, white spruce (*Picea glauca*) has been known as a plastic species because of its wide distribution across North America and its ability to recolonize areas at the end of glaciation (Nienstaedt and Zasada, 1990). It can support extremely diverse site conditions in terms of temperature, moisture, light exposure or soil type (Sutton, 1973). White spruce seedlings were sporadically found regenerating on the Sigma-Lamaque gold mine tailings at Val d’Or, Abitibi, Quebec. This rocky soil is essentially made of biotite, an iron-rich mica (Taner et al., 1986). While considered at low risk for contamination (Beauregard et al., 2012), the mine waste material – a pile of coarse rocks and a basin full of fine ground tailings –, constitute a rather hostile environment for vegetation establishment because of its unbalanced mineral content, alkaline pH, poor soil structure, and water holding capacity of the substrate. However, the rhizosphere of the healthy seedlings found on site revealed a community of fungi that is distinct from the adjacent nursery, forest edge, and natural forest ecosystems (Nadeau and Khasa, 2016).

By expanding the surface absorption of their hosts, fungi have the ability to increase water uptake and to extract insoluble forms of nutrients otherwise unavailable to the plant host (Balogh-Brunstad et al., 2008). Mycorrhizal associations have a tremendous potential to be used for land reclamation albeit some species and strains may be better adapted than others to the extreme site conditions of the Sigma-Lamaque mine tailings (Callender et al., 2016). Likewise, nitrogen-fixing bacteria have been found in the soil of all ecosystems including the boreal forest (Marshall, 2000). The inoculation of plant roots with plant growth promoting rhizobacteria (PGPR) of different genera including *Acetobacter, Agrobacterium, Bacillus, Burkholderia, Mycobacterium, Pseudomonas, Rhizobium, Sphingomonas*, and *Staphylococcus* has previously been shown to increase mineral accessibility to plants (reviewed in Rodríguez and Fraga, 1999; Tilak et al., 2005; Uroz et al., 2009; Dynarski and Houlton, 2018). For instance, *Pseudomonas* and *Bacillus* strains associated with maize roots of the Himalayan mountains showed marked P-solubilization activity (Zahid et al., 2015). Similarly, *Burkholderia glathei* inoculated on Scots pine roots (*Pinus sylvestris*) significantly increased plant Mg and K uptake from biotite substrate (Calvaruso et al., 2006). But in all these studies, the benefits conferred by the microsymbiotic association are unequal and site-, strain-, and/or plant specific. Although numerous reports list mycorrhizal fungi and PGPR that have been screened for their potential as biofertilizers in agriculture (see most recent references: Amin and Latif, 2016; Sritongon et al., 2017; Zahoor et al., 2017), the information on spruce trees interacting with a combination of fungal and bacterial symbionts under harsh conditions such as abandoned mine tailings of the Canadian north has never been studied before. In order to develop an efficient microbial consortium, it is necessary to test different microorganisms for the challenging stress and the synergistic interactions between fungi and bacteria in a way to form a basis of a cumulative impact on plant establishment.

Thus, to rapidly reforest the mining site, the main objective of the study was to assess the efficiency of selected allochthonous and indigenous mycorrhizal and bacterial symbionts in promoting adequate plant nutrition and in limiting toxicity of such an unfavorable substrate. A greenhouse experiment was conducted in which the establishment of white spruce seedlings on waste rocks or fine tailings of Sigma-Lamaque gold mine was evaluated. Both root and foliar nutrient concentrations (N, P, K, Ca, Mg, Fe) of white spruce seedlings were recorded after 32 weeks of growth during which seedling survival, health and growth parameters were also monitored (Nadeau et al., 2018). Results were comprehensively analyzed to refine our working hypothesis that favors the use of well-adapted microsymbionts directly isolated from the mining site over allochthonous ones.

## Materials and Methods

### Experimental Design and Treatments

Detailed descriptions of the experimental design, treatments, growth and health measurements are presented in the companion paper (Nadeau et al., 2018).

Briefly, tailings collected directly from Sigma-Lamaque gold mine were used for this glasshouse trial. Three-week-old white spruce seedlings were grown in waste rocks or fine tailings and inoculated with or without a mycorrhizal fungus isolated from healthy naturally regenerating white spruce seedlings on Sigma-Lamaque gold mine coarse tailings (Nadeau and Khasa, 2016; Nadeau et al., 2016): *Tricholoma scalpturatum* Tri. scalp. MBN0213 GenBank #KC840613 or *Cadophora finlandia* Cad. fin. MBN0213 GenBank #KC840625*;* or *Hebeloma crustuliniforme* UAMH5247 from the Centre for Forest Research genomic and microbial collections^1^ and with or without nitrogen-fixing bacteria [*Pseudomonas putida* MBN0213 GenBank #AY391278, *Rhizobium radiobacter* MBN0213 GenBank #FR828334 isolated from the mining site; or *Azotobacter chroococcum* ATCC 9043 purchased from CEDARLANE Laboratories Ltd. (Ontario, Canada)]. The experiment was run for 32 weeks in a glasshouse at the Université Laval under semi-controlled conditions.

The study was designed as a randomized complete block (RCB) with three crossed fixed factors (*Tailing type* × *ECM fungi* × *PGPR*). There were 32 treatments including the controls with three replicates randomly placed within each of the four blocks for a total of 384 experimental units consisting of a 1.75 L pot containing one white spruce seedling (**Supplementary Figure S1**).

### Measurements of Seedling Health and Growth

Detailed description of growth and health measurements are presented in the companion paper (Nadeau et al., 2018). Briefly, we used a portable fluorometer PAM-2000 with the data acquisition software DA-2000 (Heinz Walz, Effeltrich, Germany) to measure photochemical efficiency, we used WinSEEDLE to determine specific surface foliar areas (SSFA) of green, yellow, brown, dark red, and light red foliar tissues. Fresh needles, stem and roots were measured, weighted, and/or observed under a microscope to calculate the level of fungal colonization. Needles, stem and roots were dried for 7 days at 65°C to calculate the percentage of water content [(Fresh weight – Dry Weight)/Fresh weight]^∗^100.

### Monitoring of Seedling Survival and Nutrient Content Analyses

Seedling survival was assessed every 4 weeks through visual observations. Seedlings were considered dead when light red needles had no green color left.

For nutrient content analysis, replicates within treatments were pooled together in each block. Seedlings roots and needles were ground separately in a Wiley Mill. Samples were digested in concentrated H_2_SO_4_ and 50% H_2_O_2_. Chemical analyses of nitrogen (N), potassium (P), phosphorus (K), magnesium (Mg), calcium (Ca), and iron (Fe) in roots and needles were performed on the digested tissues following techniques outlined in Kalra (1998) and Quoreshi and Khasa (2008). Other micronutrients were not measured because their concentrations in tailings were neither a limiting nor a toxic factor.

### Statistical Analyses

#### Differences Among Treatments

As described in the compagnion paper (Nadeau et al., 2018), all the statistical analyses were conducted with the SAS software (SAS Institute Inc., 2012). Survival data were quantified in percentage and compared using χ^2^ test with PROC FREQ. Nutrient data were subjected to three-way analyses of variance (tailing type × fungi × bacteria) using PROC GLM. No transformation was necessary for root N, P, K, Ca, and Mg concentrations, and foliar K, Ca, and Mg concentrations. Log transformations were performed with root Fe concentration, and foliar N, P, and Fe concentrations.

Significance for all analyses was set at α = 0.05 (*P* ≤ 0.05). Means and standard errors of each treatment were calculated for all variables.

#### Correlation Analyses

Correlations between the percentage of roots colonized by fungi and nutrient concentration variables were investigated using PROC CORR. Correlation analyses between health variables (photochemical efficiency, percentage of healthy green foliage, and percentage of dark red foliage), growth variables (root, stem, and needle dry biomass), and nutritional variables (N, P, K, Mg, Ca, and Fe concentrations in roots and foliage) were performed in order to determine which soil elements and concentration affected positively or negatively seedling health and growth. For these analyses, block means per treatment were used. Significance for all Pearson correlation coefficients (*r*) was set at α = 0.05 (*P* ≤ 0.05).

## Results

### Plant Establishment on Mine Tailings

**FIGURE 1:**
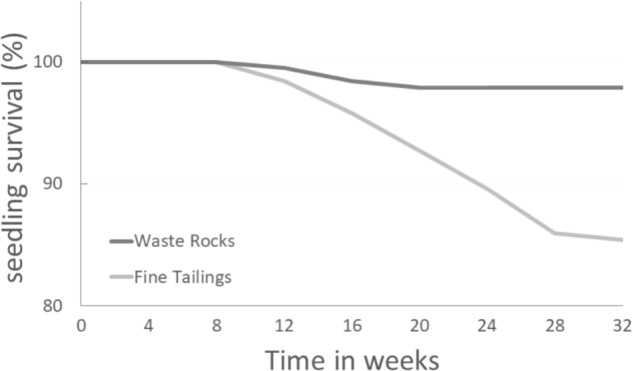
White spruce seedling survival percentage over time on two different gold mine tailing types. White spruce seedlings were grown for 32 weeks on waste rocks (WT) or fine tailings (FT). Seedling survival rates (%) were monitored and compared. After 12, 16, 20, 24, 28, and 32 weeks, the *p*-values were 0.5622, 0.1261, 0.0158, 0.0007, <0.0001, and <0.0001 (Chi-square test; α = 0.05), respectively.

Seedling mortality on both soil types began 8 weeks after the experiment started. While seedling mortality stabilized during the 16th week for the waste rock treatment, on fine tailings the percentage of seedling survival stopped decreasing during the 28th week only (**Figure 1**). Difference started to be significant after 20 weeks (*p*-value = 0.0158).

Most of the growth and health-related variables measured were independently affected by tailing type in the same manner (**Table 1**). Seedlings grew much faster and healthier on waste rocks than on fine tailings. Aboveground, measured parameters diverged by up to 20% between the two treatments. Needle biomass, stem biomass and stem length were significantly higher for seedlings planted on waste rocks than those on fine tailings (all *p*-values < 0.0001). Belowground, the root system also benefited more from waste rocks tailing. Root biomass, root length, root area and root volume data of plants grown on waste rocks were 12%, 13%, 15%, and 17% higher, respectively, than those grown on fine tailings (all *p*-values < 0.001).

**Table 1:** White spruce seedlings growth and health-related parameters affected by tailing type.

	Waste rocks	Fine tailings
Needle dry biomass (mg)	33.9 ± 0.8^a^	27.2 ± 0.9^b^
Stem dry biomass (mg)	7.9 ± 0.2^a^	6.6 ± 0.2^b^
Stem length (mm)	24.2 ± 0.3^a^	21.7 ± 0.3^b^
Root dry biomass (mg)	51 ± 1.4^a^	44.7 ± 1.4^b^
Total root length (mm)	214.2 ± 5^a^	186 ± 4.9^b^
Total root area (mm^2^)	29.2 ± 0.8^a^	24.7 ± 0.7^b^
Total root volume (mm^3^)	0.32 ± 0.01^a^	0.27 ± 0.01^b^
Dark red foliage (%)	13.9 ± 1.3^b^	18.1 ± 1.4^a^
Root water content (%)	77.6 ± 0.4^a^	76.2 ± 0.4^b^
Shoot water content (%)	48.4 ± 0.3^b^	51 ± 0.3^a^


*White spruce seedlings were grown for 32 weeks on waste rocks (WT) or fine tailings (FT). Belowground ground, root dry biomass (mg), root volume (mm^*3*^) and total root length (cm) were affected by tailing type treatment. Aboveground, growth-related parameters affected by tailing type treatment were needle dry biomass (mg), stem dry biomass (mg) and stem length (mm). Proportion of dark red foliage (%) and root water content (%) were affected by tailing type treatment. Values are means ± SE. Different letters indicate significant difference for each growth and health-related parameter between waste rocks and fine tailings.*

In agreement with survival rate and growth data, we found that plant health was also affected by tailing type. The percentage of dark red foliage (a bronzing symptom that can be associated with toxic mineral concentration) was much lower in seedlings grown on coarse waste rocks than on fine tailings (*p*-value < 0.0001). Plant-water status was also improved for seedlings planted on waste rocks than for seedlings planted on fine tailings that exhibit a more conservative water-balance. On fine tailings, root water content was lower (*p*-value = 0.0278) and plants strictly maintained higher shoot water content (*p*-value < 0.0001) than on coarse waste rocks.

### Influence of Mineral Uptake on Plant Physiology

Among the above-mentioned health and growth parameters, several were correlated with nutrient concentrations that could diverge widely in this experiment from concentration ranges encountered in perfectly healthy seedlings. This is particularly true for Ca and Fe for which concentrations were systematically recorded at levels exceeding the normal level by up to seven and 40 times but also for other elements [**Table 2**, measured concentration ranges diverging from the ones observed in healthy seedlings (Van den Driessche, 1991) are indicated in bold].

**Table 2:** Pearson correlation coefficient (*r*) and their associated *p*-values calculated for physiological parameters and the measured root and needle nutrition variables.

		Root concentration						Needle concentration					
		
		N	P	K	Ca	Mg	Fe	N	P	K	Ca	Mg	Fe
Concentration range (g/kg) observed in healthy seedlings (Van den Driessche, 1991)		12–15	1–2	3–10	2–5	1–2	5.10^-3^–0.01	12–15	1–2	3–10	2–5	1–2	5.10^-3^–0.01
Concentration range (g/kg) measured		**9**–**28**	0.5–2	3–9	**10**–**20**	**2**–**5**	**0.3**–**4**	**7**–**40**	0.4–3	**3**–**16**	**10**–**34**	1–2.8	**0.1**–**1**
Root dry	r	**-0.53**	**0.19**	0.11	**-0.63**	0.11	**-0.26**	**-0.2**	0.04	**-0.26**	**-**0.1	**-**0.12	**-0.19**
biomass	*p-valu*e	**<0.0001**	**<0.05**	0.2	**<0.0001**	0.2	**<0.01**	**<0.05**	0.6	**<0.01**	0.3	0.2	**<0.05**
Stem dry	r	**-0.44**	0.1	**-**0.06	**-0.58**	0.06	**-0.22**	**-0.23**	**-**0.04	**-0.24**	**-0.3**	**-0.18**	**-**0.14
biomass	*p-valu*e	**<0.0001**	0.3	0.5	**<0.0001**	0.5	**<0.05**	**<0.05**	0.6	**<0.01**	**<0.001**	**<0.05**	0.1
Needle dry	r	**-0.49**	0.03	**-**0.16	**-0.54**	0.01	**-0.25**	**-0.38**	**-**0.16	**-0.25**	**-0.59**	**-0.35**	**-0.18**
biomass	*p-valu*e	**<0.0001**	0.7	0.1	**<0.0001**	0.9	**<0.01**	**<0.0001**	0.1	**<0.01**	**<0.0001**	**<0.0001**	**<0.05**
Total root length	r	**-0.51**	**0.29**	0.05	**-0.65**	0.05	**-0.48**	**-0.35**	0.17	**-0.32**	**-**0.04	**-**0.02	**-0.53**
	*p-valu*e	**<0.0001**	**<0.001**	0.6	**<0.0001**	0.6	**<0.0001**	**<0.0001**	0.05	**<0.001**	0.7	0.8	**<0.0001**
Plant height	r	**-0.48**	0.17	**-**0.13	**-0.62**	0.05	**-0.33**	**-0.43**	0.00	**-0.33**	**-0.39**	**-0.19**	**-0.52**
	*p-valu*e	**<0.0001**	0.06	0.2	**<0.0001**	0.6	**<0.001**	**c**	0.9	**<0.001**	**<0.0001**	**<0.05**	**<0.0001**
Dark red	r	**0.28**	**-**0.15	**-0.19**	**0.47**	**-**0.06	**0.26**	0.01	**-**0.13	**0.26**	**-**0.15	0.12	**0.21**
foliage (%)	*p-valu*e	**0.0011**	0.0937	**0.0347**	**<0.0001**	0.4841	**0.0036**	0.8893	0.1435	**0.0035**	0.0903	0.1691	**0.0197**
Healthy green	*r*	**-0.2**	0.16	**0.24**	**-0.42**	0.1	**-0.19**	0.11	**0.23**	**-0.23**	**-0.21**	**-**0.13	**-**0.12
foliage (%)	*p-valu*e	**0.02520.28**	0.0627**-**0.15	**0.0071-0.19**	**<0.00010.47**	0.2487**-**0.06	**0.03260.26**	0.22320.01	**0.0095-**0.13	**0.00860.26**	**0.0198-**0.15	0.14160.12	0.0197**0.21**
Dark red foliage (%)	*p-valu*e	**0.0011**	0.0937	**0.0347**	**<0.0001**	0.4841	**0.0036**	0.8893	0.1435	**0.0035**	0.0903	0.1691	**0.0197**


*Optimal and measured concentration ranges are given for indicative purposes. Measured concentration ranges above normal as well as significant correlations coefficients at α ≤ 0.05 are in bold.*

Growth parameters were negatively correlated with increased concentrations of Ca and N encountered in seedling roots and with concentrations of root Fe, and K, and with N in needles. Negative correlations were also observed between root dry biomass and needle Fe concentration; between stem dry biomass and needle Ca and Mg concentrations; between needle dry biomass and the needle concentrations of Ca, Mg and Fe. For health-related parameters, the percentage of dark red foliage was positively correlated with increasing concentrations of nutrients in roots (Ca, N, and Fe) and needles (K, Fe); it was negatively correlated with root K concentration. Conversely, the percentage of healthy green foliage was positively correlated with an increased root K concentration and negatively correlated with the concentration of Ca, N, and Fe in roots and with needle K and Ca concentrations.

The strongest relations of correlation were observed for both growth and health-related parameters with N and Ca concentrations. The negative correlations between N concentration in roots with root, stem and needle dry biomass were of moderate strength (-0.53 ≤ *r* ≤ -0.44). The negative coefficients of correlation between dry biomass and Ca concentrations in roots and in needles were especially strong (*r* = -0.63, *p*-value ≤ 0.0001 and = -0.59, *p*-value ≤ 0.0001, respectively). Finally, root Ca concentration did also affect at a slightly more moderate level the percentage of dark red foliage (*r* = 0.47, *p*-value ≤ 0.0001) and of healthy green foliage (*r* = -0.42, *p*-value ≤ 0.0001).

### A Better Nutrition Status for Plants Grown on Coarse Waste Rocks

**FIGURE 2:**
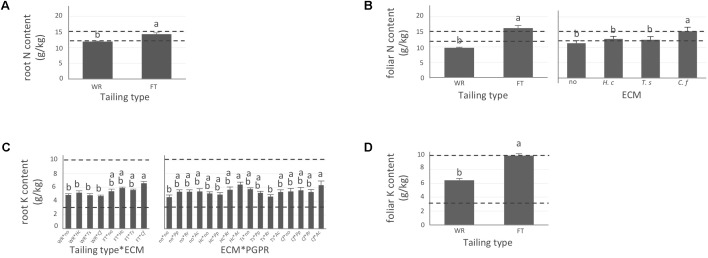
Nitrogen and potassium concentrations of white spruce roots and needles. White spruce seedlings were grown for 32 weeks on waste rocks (WT) or fine tailings (FT) with or without mycorrhizal fungi inoculation (noF = no fungus, +*Hc* = *Hebeloma crustuliniforme*, +*Ts* = *Tricholoma scalpturatum*, and +*Cf* = *Cadophora finlandia*) and with or without inoculation of rhizobacteria (noB = no bacteria, +*Pp* = *Pseudomonas putida*, +*Rr* = *Rhizobium radiobacter*, and +*Ac* = *Azotobacter chroococcum*). **(A)** Root nitrogen (N) content (g/kg) was affected by tailing type treatment (*p*-value < 0.0001); **(B)** Foliar N content was independently affected by tailing type and fungal association (*p*-values < 0.0001). **(C)** For root potassium (K) concentration (g/kg), there was an interaction between the two factors “Tailing type x fungi” (*p*-value = 0.0112) and between the two factors “fungi x Bacteria” (*p*-value = 0.0358); **(D)** foliar K concentration was affected by tailing type (*p*-value < 0.0001). Dotted lines indicate optimal concentration ranges (Van den Driessche, 1991). Values are means ± SE. Different letters indicate significant difference.

**FIGURE 3:**
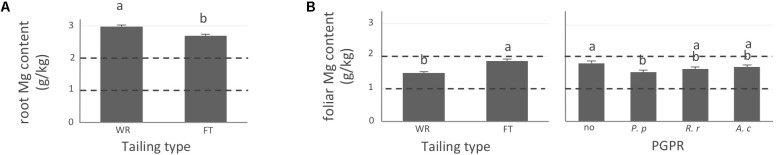
Magnesium concentration of white spruce roots and needles. White spruce seedlings were grown for 32 weeks on waste rocks (WT) or fine tailings (FT) with or without mycorrhizal fungi inoculation (noF = no fungus, +*Hc* = *Hebeloma crustuliniforme*, +*Ts* = *Tricholoma scalpturatum*, and +*Cf* = *Cadophora finlandia*) and with or without inoculation of rhizobacteria (noB = no bacteria, +*Pp* = *Pseudomonas putida*, +*Rr* = *Rhizobium radiobacter*, and +*Ac* = *Azotobacter chroococcum*). **(A)** Root magnesium (Mg) content (g/kg) was affected by tailing type treatment (*p*-value < 0.0001); **(B)** foliar Mg content was independently affected by tailing type and by bacterial association (*p*-values; <0.0001 and = 0.0058). Dotted lines indicate optimal concentration ranges (Van den Driessche, 1991). Values are means ± SE. Different letters indicate significant difference.

**FIGURE 4:**
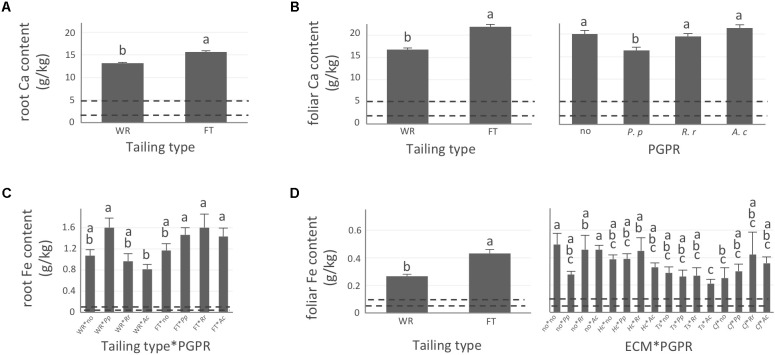
Calcium and iron concentrations of white spruce roots and needles. White spruce seedlings were grown for 32 weeks on waste rocks (WT) or fine tailings (FT) with or without mycorrhizal fungi inoculation (noF = no fungus, +*Hc* = *Hebeloma crustuliniforme*, +*Ts* = *Tricholoma scalpturatum*, and +*Cf* = *Cadophora finlandia*) and with or without inoculation of rhizobacteria (noB = no bacteria, +*Pp* = *Pseudomonas putida*, +*Rr* = *Rhizobium radiobacter*, and +*Ac* = *Azotobacter chroococcum*). **(A)** Root calcium (Ca) content (g/kg) was affected by tailing type treatment (*p*-value < 0.0001); **(B)** Foliar Ca concentration was independently affected by tailing type and by bacterial association (*p*-values < 0.0001); **(C)** for root iron (Fe) concentration (g/kg), there was an interaction between the two factors “Tailing type x bacteria” (*p*-value = 0.0206); **(D)** Foliar Fe concentration was affected by tailing type treatment (*p*-value < 0.0001), there was an interaction between the two factors “fungi × bacteria” (*p*-value = 0.0472). Dotted lines indicate optimal concentration ranges (Van den Driessche, 1991). Values are means ± SE. Different letters indicate significant difference.

Apart from P for which concentration did not vary significantly during the experiment and remained only slightly below or within normal concentration range observed in healthy coniferous seedlings (**Supplementary Figure S2**, optimum concentration range indicated by the two dashed lines), tailing type influenced all tested element concentrations in roots and/or needles (**Figures 2–4**). For all elements, maxima were systematically measured in roots of plants grown on fine tailings but not on coarse waste rocks.

In general, N concentration was only slightly below optimum and benefited significantly from fine tailing treatment in both roots and needles (**Figures 2A,B**, *p*-values < 0.0001) where it exceeded a normal concentration range. A similar pattern was observed for K. In roots, values were within normal ranges (**Figure 2C**; *p*-value = 0.0112) and reached a maximum in needles of seedlings growing on fine tailings (**Figure 2D**; *p*-value < 0.0001). Soil Mg concentration was high and seedling root uptake was higher than optimum on both types of soil but particularly on coarse waste rocks (**Figure 3A**; *p*-value < 0.0001). But because translocation factor (**Supplementary Figure S3**) was also much lower on waste rocks, it resulted in an opposite pattern in needles: Mg concentration was normal and even lower than in fine talings-grown plants (**Figure 3B**; *p*-value < 0.0001). The concentrations of Ca and Fe were especially high (**Figure 4**). Compared to those growing on fine tailings, seedlings growing on waste rocks exhibited lower concentrations of Ca in roots and needles (**Figures 4A,B**; *p*-values < 0.0001). The aboveground concentration of Ca differed by 23% in seedlings planted on waste rocks compared to seedlings on fine tailings. A similar pattern was observed for Fe in roots (in interaction with bacterial factor, **Figure 4C**; *p*-values = 0.0206) and in needles (**Figure 4D**; *p*-value < 0.0001) where concentration was 38% lower in plants grown on waste rocks and dropped clearly compared to root concentration (compare *y*-axis scale ranges, **Figures 4C,D**).

In summary, the nutritional status of plants grown on coarse waste rocks was in general significantly better than those grown in fine tailings. It was closer or within concentration range of healthy white spruce seedlings for N and K, the availability of which was limited in Sigma-Lamaque gold mine tailings; Mg concentration fell within normal concentration range in needles; while Ca and Fe concentrations were indeed above optima but at significantly lower levels compared to plants grown on fine tailings.

### Influence of Symbiotic Association on Plant Nutrition

As shown in the companion paper (Nadeau et al., 2018), root mycorrhization rate was also greater on waste rocks than on fine tailings, and much higher when plants were inoculated with the fungus *C*. *finlandia* originally isolated from the mining site. Albeit the concentrations of only a few elements were influenced by mycorrhizal inoculation, *C*. *finlandia* is a good ectomycorrhizal fungal candidate: only plants inoculated with *C. Finlandia* differ from controls regarding foliar N concentration (**Figure 2B**), root K concentration (**Figure 2C**) and foliar Fe content (**Figure 4D**).

The percentage of root tips colonized by *H*. *crustuliniforme* had a negative effect on root N concentration (**Table 3**) and was positively correlated with root P concentration (as it was for plants colonized with *T*. *scalpturatum*). On fine tailings only, the association with *C*. *finlandia* resulted in a positive translocation factor from the roots to the needles (**Supplementary Figure S3**). *C*. *finlandia* was also the one symbiont for which the negative correlation between root mycorrhization rate and root concentration of Ca was the strongest (*r* = 0.66, *p*-value ≤ 0.0001). It was the only one for which root mycorrhization rate was negatively correlated with root Fe concentration: the more roots were colonized the least Ca and Fe they uptake from a soil where both are found in excess. However, in roots, none of the above-mentioned nutrients was influenced by fungal treatment. Both fungi and rhizobacteria affected the aboveground Fe concentration. There was an interaction between the two treatments (**Figure 4D**; *p*-value = 0.0472). Compared to seedlings without symbionts, inoculation with either *C*. *finlandia* alone or with both the fungus *T*. *scalpturatum* and the rhizobacterial strain *A*. *chroococcum* reduced by half the accumulation of Fe in needles while no other associations improved foliar plant Fe status.

**Table 3:** Pearson correlation coefficient (*r*) and their associated *p*-values calculated for the proportion of root tips colonized by different fungi and measured root and needle nutrition variables.

		Root content						Needle content					
		
		N	P	K	Ca	Mg	Fe	N	P	K	Ca	Mg	Fe
*Cadophora*	*r*	**-**0.3	0.28	**-**0.21	**-0.66**	0.14	**-0.44**	**-**0.32	0.05	**-0.43**	**-**0.11	**-**0.12	**-**0.09
*finlandia*	*p-valu*e	0.0942	0.1156	0.2426	**<0.0001**	0.4354	**0.0109**	0.0764	0.7959	**0.0137**	0.5308	0.5217	0.6092
*Tricholoma*	*r*	**-**0.21	**0.42**	0.05	**-0.49**	0.23	**-**0.23	0.04	**-**0.02	**-**0.15	0.25	**-**0.11	**-**0.3
*scalpturatum*	*p-valu*e	0.2448	**0.0175**	0.7757	**0.0047**	0.2048	0.207	0.8268	0.8905	0.4071	0.1705	0.5419	0.0966
*Hebeloma*	*r*	**-0.49**	**0.39**	0.26	**-0.49**	0.34	**-**0.23	**-**0.2	0.33	**-**0.17	**-**0.22	**-**0.27	**-**0.29
*crustuliniforme*	*p-valu*e	**0.0042**	**0.0283**	0.1567	**0.0047**	0.0594	0.1957	0.2744	0.0661	0.3384	0.2369	0.1357	0.1097


*Significant correlations coefficients at α ≤ 0.05 are in bold.*

Indeed, association with *A*. *chroococcum* alone resulted in the highest foliar Fe concentration. *R*. *radiobacter* treatment also had a negative impact on foliar Fe concentration (systematically > 0.4 g/kg except in combination with *T*. *scalpturatum*). Besides foliar Fe measurements, it also had a significant impact on root K, foliar Mg, foliar Ca, and root Fe concentrations. On waste rocks, *A*. *chroococcum* clearly benefitted white spruces reducing by half root Fe concentration (**Figure 4C**, *p*-value = 0.0206) but the same strain had no effect on fine tailings. In the interaction with fungus, the bacterial strain also influenced K concentration in roots. Values remained within the normal range in all cases, but plants inoculated with *A*. *chroococcum* exhibited higher K concentrations (**Figure 2C**; *p*-value = 0.0358), a minimum average K concentration was observed for plants associated with both *T*. *scalpturatum* fungus and *R*. *radiobacter* bacterium. At the foliar level, Mg and Ca concentrations (**Figures 3B, 4B**, *p*-values = 0.0058 and < 0.0001, respectively) were reduced by *P*. *putida* inoculation, while plants treated with *A*. *chroococcum* exhibited concentrations similar to those of plants treated with *R*. *radiobacter* or to non-inoculated white spruce seedlings. To conclude, the impact of rhizobacteria factor on overall plant nutritional status is complex: *A*. *chroococcum* improved Fe nutrition in some cases, *P*. *putida* improved both foliar Mg and Ca concentrations, and *R*. *radiobacter* was the only tested strain with no positive effect.

## Discussion

The results showed the substantial effect of microsymbiont inoculation on white spruce seedlings growing on Sigma-Lamaque gold mine tailings. In agreement with our main hypothesis, the nutritional status of plants associated with native strains of fungi and rhizobacteria was significantly improved.

### Effect of Tailing Type and Particle Size on Plant Performance

In this experiment, seedlings performed much better on waste rocks than on fine tailings with respect to survival rate, health and growth. On coarse waste rocks, plants also tend to a more balanced mineral content.

Tailings do not contain any nitrogen source and only small concentrations of phosphorus and potassium. Thus one may think macro-elements availability is the limiting factor for plant survival on the mining site. However, increasing concentrations of N, P, or K in plant tissue were not associated with improvements in seedling health and growth. On the contrary, high root and foliar N concentrations were negatively correlated with seedling biomass or percentage of healthy green foliage. While macro-element concentrations in plants grown on waste rocks were within or only slightly below normal concentration ranges, N and K were measured at significantly higher levels in plants on fine tailings, mineral weathering being normally higher in finer particle substrates (Modak et al., 2001). In some cases, N concentration actually exceeded by more than twice the maximum observed in healthy coniferous trees (Van den Driessche, 1991).

Likewise, the concentrations of calcium, magnesium and iron – found in abundance in the substrates – were in general lower in roots and/or needles of plants grown on fine tailings than on waste rocks. Ca and Fe concentrations were systematically present at levels largely above the normal concentration ranges. Indeed, in this study, correlation analyses highlight the negative impact of excessive mineral uptake (Ca and Fe but also N) on plant growth- and health-related parameters. Because minerals are less mobile in coarser size residues, white spruce seedlings may perform better on waste rocks than on fine tailings of the Sigma-Lamaque gold mine – where high element concentration rather than nutrient deficiency could limit plant establishment and consequently the development of an effective phytorestoration program.

### Allochthonous Microsymbionts Fail to Improve Plant NPK Nutrition

In our study, microsymbionts played an important role in reducing Ca and Fe level but the concentrations of most other elements and especially the primary macro-elements N–P–K, were not improved by the different inoculants. Microsymbiotic benefits are often specific to the site, the strain or even to the host involved. For instance regarding nitrogen nutrition, the ability of *Azotobacter* sp. to fix nitrogen is highly dependant on phosphate availability (Brown et al., 1962; Dynarski and Houlton, 2018); not all strains of *Rhizobium radiobacter* are capable of fixing nitrogen (Humphry et al., 2007); and the fungus *C*. *finlandia* was found important for N nutrition of Norway spruce (*Picea abies*, Mrnka et al., 2009) but not for scots pine (*Pinus sylvestris*, Alberton et al., 2010). Regarding phosphorus nutrition, foliar P does not usually differ between mycorrhizal and non-mycorrhizal seedlings, although hyphae are known to be more effective than roots at P uptake (Smith and Hinckley, 1995). Adeleke et al. (2012) also found that the inoculation of seedlings with ECM fungi enhanced potassium mobilization but did not increase foliar K concentration.

In our experiment, none of the bacterial treatments allowed the increase of seedling N uptake. Only foliar N concentration was significantly increased by the association with the fungus *C*. *finlandia*, albeit other treatments, including the one without symbionts, fall within normal concentration range. Because the tailings were not sterilized to mimic field conditions, we suspect that some native diazotrophic bacteria may have already been present (Dynarski and Houlton, 2018). The high pH of the substrate makes it a favorable environment (Brown et al., 1962) for the growth and development of several species capable of improving plant N nutrition (Damir et al., 2011). Interestingly, when an excessive concentration of nitrogen was measured (associated with disturbance of seedling health and growth), it was often in non-inoculated plants or in plants inoculated with allochthonous microsymbionts. In the context of multi-partite interactions like the ones we established or more complex ones that could occur in the rhizosphere of trees planted on the mine tailings, N-fixing free-living bacteria naturally occurring *in situ* may coexist better with inoculated fungi or rhizobacteria originating from the same ecosystem (Uroz et al., 2007). Fungi and bacteria already present in the mine tailings (Nadeau and Khasa, 2016) could outcompete microsymbionts that are not well-adapted, hence limiting plant N nutrition and the successful revegetation with allochtonous microsymbionts.

The tested microsymbiotic associations also failed to clearly improve P and K concentrations in roots where they were measured under (for P) or within normal concentration range (for P and K). Detailed analysis of the data helped us to refine our strategy and to consider, in addition to mycorrhizal fungi inoculation, the use of phosphorus amendment otherwise the only macro-element present in limited quantities in the substrate and within the plant tissues. Indeed, an increasing concentration of P in plant was shown to benefit seedling growth and health, and also correlated with *T*. *scalpturatum* and *H*. *crustuliniforme* root colonization rate but not with *C*. *finlandia*, a fungus yet well adapted to the mining site.

### Selection of Native Microsymbiotic Partners for the Successful Alleviation of Element Deleterious Effects

Among nutrients investigated, plant Ca concentration displayed the strongest negative correlation relationships with general seedling growth and health, followed by iron.

Regarding Mg concentration (measured at a high level in the substrate), on average, it was effectively found above normal concentration range in roots but not in needles. In needles, high levels of Mg impact growth and may lead to premature fall (Marschner, 1995).

Calcium, also found in excess in the mine tailings, is incidentally known to alleviate Mg toxicity (Brooks, 1987; Chiarucci et al., 1998). Moreover, the concentrations of both Mg and Ca in needles were low in presence of the bacterial strain *Pseudomonas putida*, which is well characterized for its ability to provide plants with iron through the massive production of siderophores (Bar-Ness et al., 1992). However, *P. putida* is a versatile species with a broad ability to several traits for adaptations (Matilla et al., 2011; Udaondo et al., 2013). The strain MBN0213 used in this study originated from the mining site. Similar to mycorrhizal fungi that produce calcium oxalate crystals to sequester toxic amounts of Ca (Snetselaar and Whitney, 1990; Arocena et al., 1999), strain MBN0213 may have developed specific mechanisms to reduce Ca toxicity.

Rhizobacteria inoculation did not clearly improve Fe distribution within plant tissues; only specific combinations resulted in low concentrations. In needles, the allochthonous *Azotobacter chroococcum* reduced Fe concentration when inoculated in consortium with *T*. *scalpturatum* but not when inoculated alone (in which case average Fe concentration equaled those found in non-inoculated seedlings or in seedlings inoculated with the siderophores-producing *P*. *putida*). In summary, *P*. *putida* MBN0213 was the most promising rhizobacterium tested. Although it neither reduced excessive Fe concentration nor improved N–P–K nutrition, it was the only bacterium that minimized Mg and Ca concentrations *in planta*, with the level of Ca being the most limiting factor we identified in this study.

Native fungi of the mining site are also of great interest, especially *C. finlandia*. It is the fungus that colonized seedling roots most efficiently and enhanced seedling health (Nadeau et al., 2018). Also, it was the only microsymbiont whose root colonization rate was negatively correlated with Fe root concentration and the most strongly correlated with Ca root uptake. Therefore, *C*. *finlandia* is capable of alleviating the deleterious effects of excessive Fe and Ca. On moderately polluted soil, the study of De Maria et al. (2011) demonstrated that an efficient microbial consortium formed by the dual inoculation of *C*. *finlandia* and PGPR enhanced goat willow (*Salix caprea*) phytoextraction efficiency. In the context of the Sigma-Lamaque gold mine tailing revegetation program, we should be able to exploit the synergistic interactions between fungi and bacteria the present study reveals.

## Author Contributions

MN and DK conceived and designed the experiments. MN performed the experiments. MN, JL, and DK analyzed the data and contributed in drafting the manuscript.

## Conflict of Interest Statement

The authors declare that the research was conducted in the absence of any commercial or financial relationships that could be construed as a potential conflict of interest.

## Footnotes

1 http://www.cef-cfr.ca/index.php?n=CEF.Collections

## Supplementary Material

FIGURE S1 Illustrations and design of the experiment. Mycorrhizal fungi and rhizobacteria used in this **(A)** glasshouse experiment were either **(B)** native from the Sigma-Lamaque gold mine site (*Tricholoma scalpturatum* (Ts), *Cadophora finlandia* (Cf); *Pseudomonas putida* (Pp), *Rhizobium radiobacter* (Rr)), or isolated from a natural forest stand (*Hebeloma crustuliniforme* (Hc)) or of commercial origin for *Azotobacter chroococcum* (Ac). White spruce saplings were either planted in **(C)** waste rocks or in fine tailings. **(D)** Randomized complete block (RCB) design with three crossed fixed factors: tailing type [fine tailing (FT) in regular font, waste rocks (WT) in bold]; fungi [none (noF) in black, Ts: green font, Cf: blue font, Hc: red font]; and bacteria [none (noB): no background, Rr: background in red, Pp: background in blue, Ac: background in green] for a total of 32 treatments, 4 blocks, 3 replicates per treatment per block, and 384 experimental units.

FIGURE S2 Phosphorus (P) concentration of white spruce roots and needles. White spruce seedlings were grown for 32 weeks on waste rocks (WT) or fine tailings (FT) with or without mycorrhizal fungi inoculation (noF = no fungus, +*H c* = *Hebeloma crustuliniforme*, +*Ts* = *Tricholoma scalpturatum*, and +*Cf* = *Cadophora finlandia*) and with or without inoculation of rhizobacteria (noB = no bacteria, +*Pp* = *Pseudomonas putida*, +*Rr* = *Rhizobium radiobacter*, and +*Ac* = *Azotobacter chroococcum*). Dotted lines indicate optimal concentration ranges (Van den Driessche, 1991). Values are means ± SE.

FIGURE S3 Translocation factors. White spruce seedlings were grown for 32 weeks on waste rocks (WT) or fine tailings (FT) with or without mycorrhizal fungi inoculation (noF = no fungus, +*Hc* = *Hebeloma crustuliniforme*, +*Ts* = *Tricholoma scalpturatum*, and +*Cf* = *Cadophora finlandia*) and with or without inoculation of rhizobacteria (noB = no bacteria, +*Pp* = *Pseudomonas putida*, +*Rr* = *Rhizobium radiobacter*, and +*Ac* = *Azotobacter chroococcum*).
