# Supplementary figures and images for: Mycorrhizae and Rhizobacteria on Precambrian Rocky Gold Mine Tailings: II. Mine-Adapted Symbionts Alleviate Soil Element Imbalance for a Better Nutritional Status of White Spruce Seedlings
Source: Front Plant Sci. 2018 Sep 3;9:1268. doi: 10.3389/fpls.2018.01268 (PMC6130232; doi:10.3389/fpls.2018.01268)

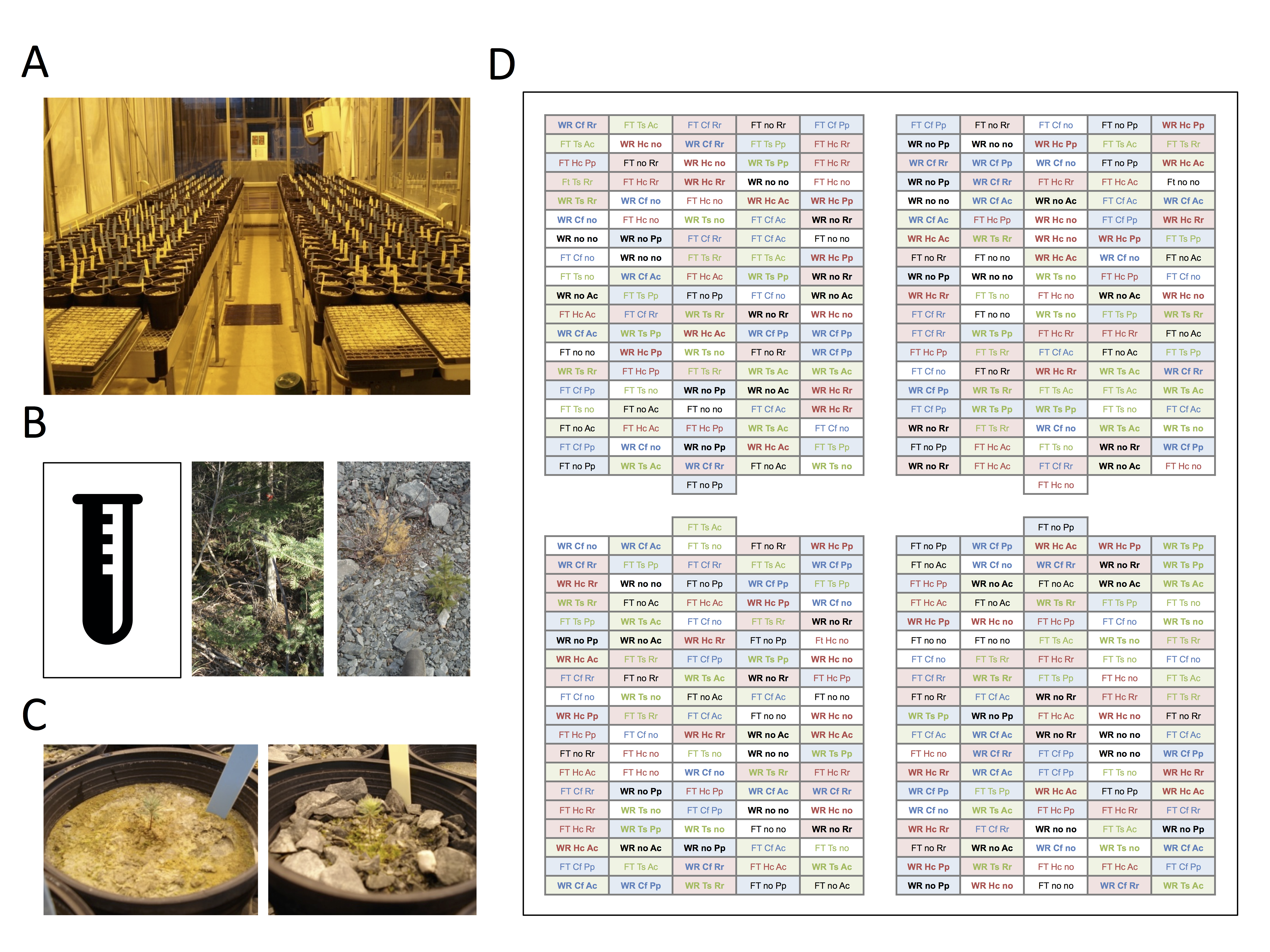

Supplement: FIGURE S1 — Illustrations and design of the experiment. Mycorrhizal fungi and rhizobacteria used in this (A) glasshouse experiment were either (B) native from the Sigma-Lamaque gold mine site (Tricholoma scalpturatum (Ts), Cadophora finlandia (Cf); Pseudomonas putida (Pp), Rhizobium radiobacter (Rr)), or isolated from a natural forest stand (Hebeloma crustuliniforme (Hc)) or of commercial origin for Azotobacter chroococcum (Ac). White spruce saplings were either planted in (C) waste rocks or in fine tailings. (D) Randomized complete block (RCB) design with three crossed fixed factors: tailing type [fine tailing (FT) in regular font, waste rocks (WT) in bold]; fungi [none (noF) in black, Ts: green font, Cf: blue font, Hc: red font]; and bacteria [none (noB): no background, Rr: background in red, Pp: background in blue, Ac: background in green] for a total of 32 treatments, 4 blocks, 3 replicates per treatment per block, and 384 experimental units. [file Image_1.TIFF]

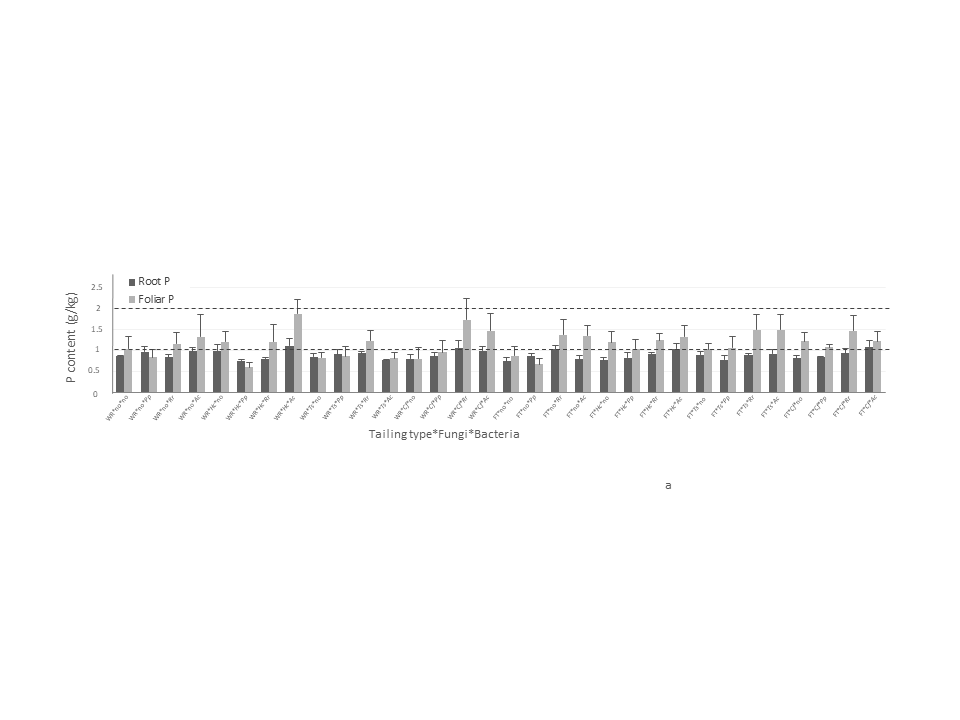

Supplement: FIGURE S2 — Phosphorus (P) concentration of white spruce roots and needles. White spruce seedlings were grown for 32 weeks on waste rocks (WT) or fine tailings (FT) with or without mycorrhizal fungi inoculation (noF = no fungus, +H c = Hebeloma crustuliniforme, +Ts = Tricholoma scalpturatum, and +Cf = Cadophora finlandia) and with or without inoculation of rhizobacteria (noB = no bacteria, +Pp = Pseudomonas putida, +Rr = Rhizobium radiobacter, and +Ac = Azotobacter chroococcum). Dotted lines indicate optimal concentration ranges (Van den Driessche, 1991). Values are means ± SE. [file Image_2.TIF]

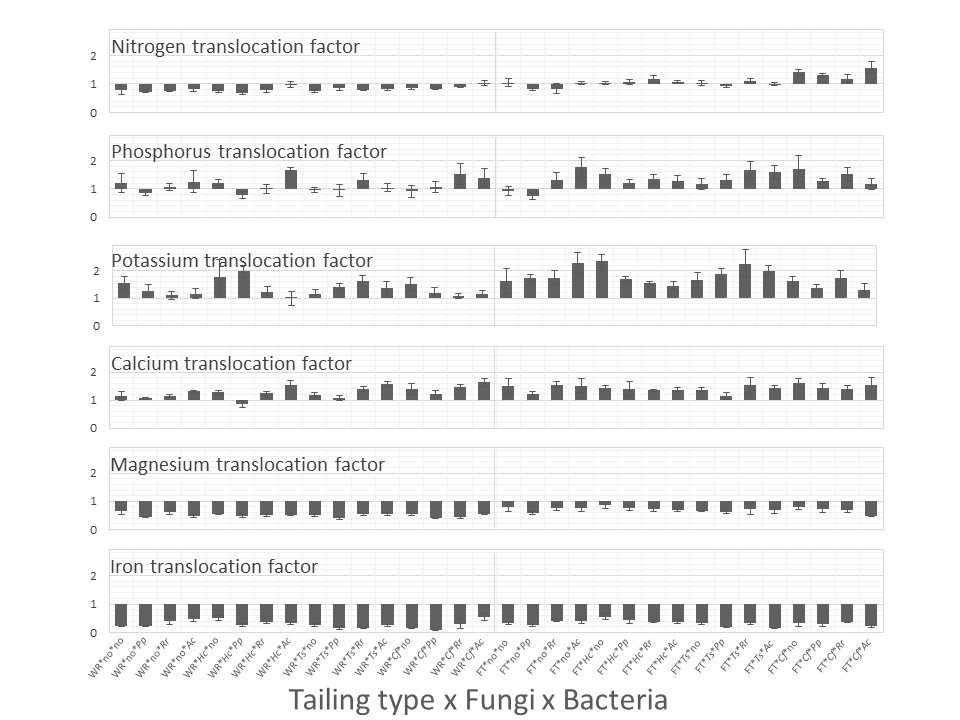

Supplement: FIGURE S3 — Translocation factors. White spruce seedlings were grown for 32 weeks on waste rocks (WT) or fine tailings (FT) with or without mycorrhizal fungi inoculation (noF = no fungus, +Hc = Hebeloma crustuliniforme, +Ts = Tricholoma scalpturatum, and +Cf = Cadophora finlandia) and with or without inoculation of rhizobacteria (noB = no bacteria, +Pp = Pseudomonas putida, +Rr = Rhizobium radiobacter, and +Ac = Azotobacter chroococcum). [file Image_3.TIF]
